# Effects of Water Level on Three Wetlands Soil Seed Banks on the Tibetan Plateau

**DOI:** 10.1371/journal.pone.0101458

**Published:** 2014-07-01

**Authors:** Miaojun Ma, Zhen Ma, Guozhen Du

**Affiliations:** State Key Laboratory of Grassland and Agro-ecosystems, School of Life Sciences, Lanzhou University, Lanzhou, P.R China; Central China Normal University, China

## Abstract

**Background:**

Although the effect of water level on germination in soil seed banks has been documented in many ecosystems, the mechanism is not fully understood, and to date no empirical studies on this subject exist. Further, no work has been done on the effect of water level on seed banks of drying and saline-alkaline wetlands in alpine areas on the Tibetan Plateau.

**Methodology:**

We examined the effects of water level (0 cm, 5 cm and 10 cm) on seed germination and seedling establishment from soil seed banks at 0–5 cm and 5–10 cm depths in typical, drying, and saline-alkaline wetlands. We also explore the potential role of soil seed bank in restoration of drying and saline-alkaline wetlands.

**Principal Findings:**

Species richness decreased with increase in water level, but there almost no change in seed density. A huge difference exists in species composition of the seed bank among different water levels in all three wetlands, especially between 0 cm and 5 cm and 0 cm and 10 cm. Similarity of species composition between seed bank and plant community was higher in 0 cm water level in drying wetland than in the other two wetlands. The similarity was much higher in 0 cm water level than in 5 cm and 10 cm water levels in all three wetlands. Species composition of the alpine wetland plant community changed significantly after drying and salinization, however, species composition of the seed bank was unchanged regardless of the environment change.

**Conclusions/Significance:**

Water level greatly affects seed bank recruitment and plant community establishment. Further, different water levels in restored habitats are likely to determine its species composition of the plant community. The seed bank is important in restoration of degraded wetlands. Successful restoration of drying and salinization wetlands could depend on the seed bank.

## Introduction

Maqu wetlands have an important ecological function and a huge influence in the entire Yellow River basin [1]–[2]. With the increase in grazing and anthropogenic disturbance, these climate-sensitive wetlands have been shrinking at a rapid rate [2]. Large areas of wetlands have been degraded by drying and salinization. Thus, restoration of this type of sensitive ecosystem is urgently needed in the Tibetan Plateau [1]–[2]. Much research has concluded that the seed bank is an important potential resource for wetland restoration [3]–[5] (e.g. Smith et al. 2002; Klimkowsk et al. 2010; Hong et al. 2012). Whether the alpine wetland seed bank has become exhausted due to the wetland drying or soil salinization is an open question.

[6] indicated that the water availability and hydrology play an important role in wetland species germination and seedling growth and are important factors that influence species composition of plant community and biodiversity [7]–[8]. Especially, the water level strongly affects wetland species germination [9]–[11]. Germination and dormancy responses of seeds might be affected by the levels of available oxygen and light in different water level [12]. Water level can influence seed germination and seedling recruitment from the seed bank [13]–[14]. Many species in the seed bank most often germinate as soon as water drawdown occurs [6], [15] and seedling became established [16]. Significantly more seeds and species germinate from seed bank in moist soil than in flooded condition [17]. Previous research assumed that the upland species, emergent species and submerged species germinate in moist soil, saturated soil and flooded conditions, respectively [18]–[19]. Much research has reported how the water regime influences flowering and seed production at the species level [20]. However, there are few studies on how species composition of the seed bank changes in different water depths at the community level. In addition, despite the fact that the effect of water level on germination of soil seed banks is documented in many ecosystems [9], [14], the mechanism is not fully understood, and to date no empirical studies on this subject exist in some sensitive habitats, especially in the alpine wetlands on the Tibetan Plateau. We hypothesized that water level is an important environmental factor in maintaining the plant community and the seed bank and that the species richness and seed density in the seed bank would decrease in 5 cm and 10 cm water levels.

Much research has investigated the correlation between the seed bank and vegetation in a variety of wetlands [9]. Some studies found that wetland seed banks have high contribution to aboveground wetland plant community regeneration [18], [21], while others found a low similarity between seed bank and plant community [18]. At present, much research focuses on exploring the relationship between the seed bank and wetland plant community in different ecosystems [1], [10]. Few studies have explored how water levels influence this relationship, no study has been done on degraded alpine wetlands on the Tibetan Plateau [2]. In a previous study, we concluded that the seed banks make a low contribution to regeneration of alpine wetland plant communities [1] but that seed banks have a high potential to contribution to restoration of saline–alkaline meadow in Tibetan Plateau [2]. However, the importance of seed bank in drying and degraded alpine wetland restoration in Tibetan Plateau is still poorly studied [1]–[2]. We hypothesized that the soil seed bank is an important resource in restoration of drying and saline-alkaline alpine wetlands on the Tibetan Plateau.

## Material and Methods

### Ethics Statement

No permits were required to carry out this study. The owner of all the study sites is P.R China. The Chinese government give us permission to conduct the study on these sites. We confirm that the field studies did not involve endangered or protected species. No vertebrate studies in this manuscript.

### Study sites

The study areas are three alpine wetlands at the Maqu Wetland Protection Area (33°45′ N, 102°04′ E) in the eastern part of the Tibetan Plateau, in Gansu Province, P.R China, with an elevation of 3200 m–3600 m above sea level. The annual rainfall is 450–780 mm and occurs during summer. Average annual temperature is 1.2°C, ranging annually from −10°C (January) to 11.7°C (July). There are 2,580 h cloud-free solar radiation and 270 frost days per year. Seed bank and aboveground vegetation sampling were carried out at three different wetlands, which are characterized as follows:

Typical wetlands (33°58′N, 101°52′E. altitude 3512 m a.s.l.). The wetland has been fenced since Oct 2005 and only grazing is done in winter by yak and Tibetan sheep. Most of the fenced wetland is covered by water only in summer to autumn (June-September), but a small part of places is flooded throughout the year. Water level ranges from 0 cm to 20 cm. Sedges (c.g. *Carex atrofusca*), grasses (e.g. *Deschampsia caespitosa*), and some wetland species (e.g. *Caltha palustris*) dominate plant community.

Drying wetlands (33°58′N, 101°52′E. altitude 3517 m a.s.l.). Under high intensity of grazing disturbance, vegetation got lower and bare ground emerged in some place, which further increased surface evaporation. No water throughout the year. The dominant species have gradually changed to alpine meadow species due to long-term overgrazing and trampling by yak and Tibetan sheep. Plant cover is 50–80%. The plant community is dominated by *Plantago asihica*, *Potentilla anserine*, *Carex moorcroftii*, *Elymus dahuricus* and *Polygonum viviparum*. Gaps created by yak and Tibetan sheep trampling (livestock) and by digging of Marmot (*Marmota himalayana*) and Tibetan Pika (*Ochotona curzoniae*) in some places.

Saline-alkaline wetlands (34°18′ N, 102°14′ E. altitude 3430 m a.s.l.). Grazing disturbance is the potential driver of the wetland drying and salinization. In some places, there large areas of saline-alkaline wetlands that result from accumulating of salts on soil surface wetland during drying. Water levels range from 0 cm to 8 cm in Autumn, and there is no water at other times of the year. The plant community is dominated by *Deschampsia caespitosa*, *Leontopodium leontopodioides*, *Potentilla anserina*, and *Sedum ulricae*. The vegetation is low and sparse in some places. Plant cover is 40–65%. There are some gaps in the vegetation created by the animals mentioned above.

### Soil seed bank sampling

Soil samples were collected in April 2012 before field seed germination, to capture both the transient and the persistent seed banks. The area of each of three wetlands was at least 200 ha. Five sites (100 m×100 m) were selected randomly in each wetland, each site as an independent spatial replicate in each of the three wetland. Selection of these sites was designed to maximize representation of each type of wetland. All sites have same orientation, aspect, exposure and annual mean temperature (1.2°C) and annual mean precipitation (620 mm). Five plots (10 m×10 m) were selected randomly in each site and 10 cylindrical soil cores (3.6 cm diameter) were sampled randomly from each plot. The soil cores were divided into 0–5 cm depth and 5–10 cm depth layers. Ten cores from each soil depth were pooled for each plot. Overall, there were 10 samples from each site (5 samples for each layer), and 50 soil samples for each habitat, a total of 150 soil samples. The area sampled was 0.25 m^2^, and soil volume was 0.03 m^3^ for each wetland.

### Treatment of soil samples and maintenance of seed trays

The germination experiment was initiated in early May 2011 to examine the floristic composition of seed bank, which was conducted in the Research Station of Alpine Meadow and Wetland Ecosystems of Lanzhou University (Hezuo Branch Station), Gansu Province, P.R China (N34°55′, E102°53′, altitude 2900 m a.s.l), also on the Tibetan plateau. The mean annual precipitation is 557.8 mm, from 2.4 mm in January to 110.3 mm in July. The mean annual temperature is 2.0°C the average temperature is 2.4°C, from −9.9°C in January to 12.8°C in July.

Soil samples from each habitat were placed in a north-facing window, and the samples were air-dried for 15 days until they could be processed. We used the seedling emergence method to examine the species composition of seed bank [22]–[23] in different water levels. The coarse debris and visible rhizomes were removed from soil samples. We divided each soil sample into three equal parts, and each soil sample was spread evenly on the top of the sterile fine sand (sterilized at 140°C for 24 h) in plastic trays (width 30 cm), each tray contained one-third of the soil sample. Three water levels (0 cm, 5 cm and 10 cm) in each wetland were manipulated to simulate three different hydrologic regimes. One-third of the trays were submerged in 5 cm of standing water, one-third in 10 cm standing water, and one-third trays in a moist soil (nonflooding) and arranged randomly in Hezuo Branch Station. The nonflooded treatment was were watered daily to keep moist (0 cm water level) and water levels in the two flooded treatment were maintained at 5 cm and 10 cm. The three water levels were maintained throughout the 5-month germination experiment from May to September. Ten control trays consisting of only sterilized sand were placed amongst the experimental trays. Seedlings were considered to be established when they produced the first true leaf. Germinated seedlings were identified and removed from the trays, unidentifiable species were grown separately until they could be indentified.

### Plant community sampling

Species composition of plant communities was surveyed during the peak of the growing season (July 2011). Ten quadrats (50 cm×50 cm) were used in typical wetland and saline-alkaline wetland and six in drying wetland. Quadrats were placed randomly within each wetlands. The presence and cover of all species were recorded within each quadrat. Cover was estimated using the Braun-Blanquet scale [24]–[26].

### Data analysis

Species richness and seed density of two soil depths (0–5 cm and 5–10 cm) per plot changes in three water level (0 cm, 5 cm and 10 cm) were compared using one-way analysis of variance (ANOVA), followed by Tukey range test. Data for each habitat were analyzed separately. Seed densities were compared using log transformed data. All ANOVA tests were conducted with a SPSS 13.0 program.

Non-metric multidimensional scaling (NMDS) was used to evaluate species composition similarity among different wetlands for seed banks and vegetation with different water levels (0, 5, and 10 cm). Data for each wetland (typical wetland, drying wetland, and saline-alkaline wetland) and all three wetlands (0 cm water level) were analyzed separately. Ordination was based on relative abundance data. Ordination was conducted using the R-program for Windows version 3.0.1, applying package VEGAN by Jari Oksanen. Similarity matrices were calculated using the Bray-Curtis coefficient.

## Results

### Vegetation changes

**Figure 1:**
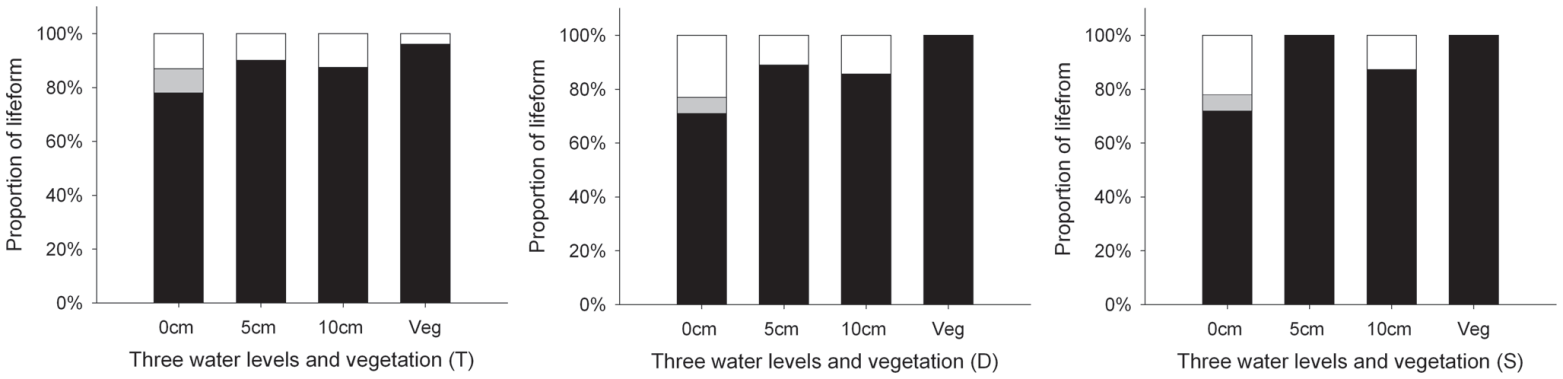
The proportion of life forms in three water levels and vegetation at different wetlands in Tibetan Plateau (Black bar: perennial species, gray bar: biennial species, and white bar: annual species). T =  Typical wetland; D =  Drying wetland; S =  Saline-alkaline wetland.

**Figure 2:**
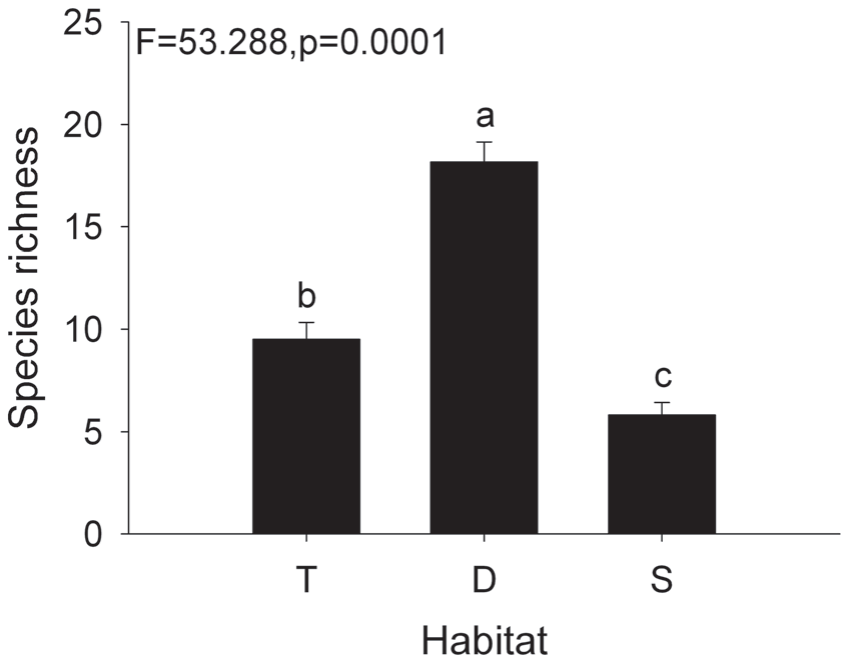
Species richness (per quadrat, mean±SE, n = 10 in habitat T and S, n = 6 in habitat D) changes in vegetation among three wetlands in the Tibetan Plateau. Letters indicate significant differences (ANOVA, Tukey range test) of mean values. T =  Typical wetland; D =  Drying wetland; S =  Saline-alkaline wetland.

**Figure 3:**
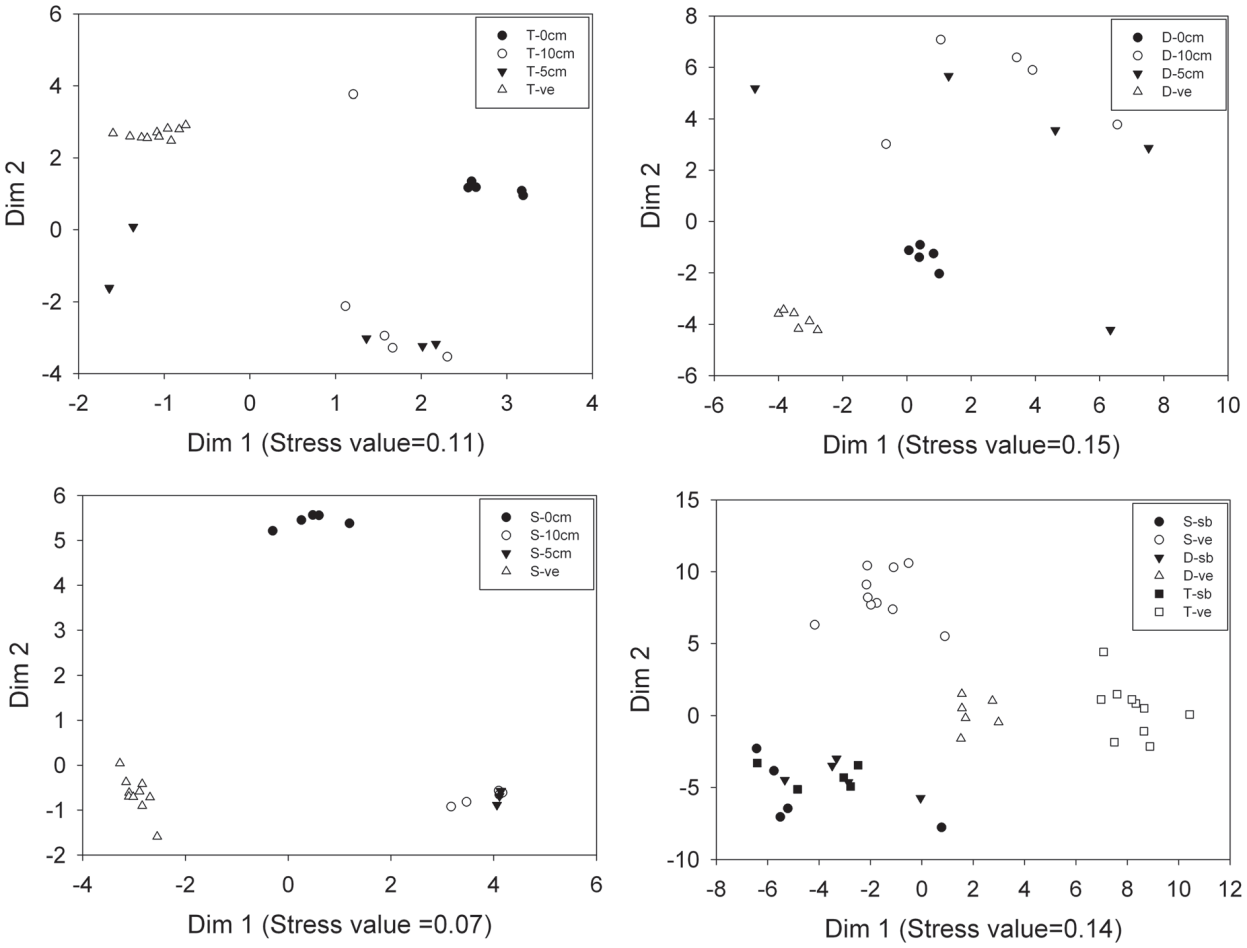
Two-dimensional nonmetric multidimensional scaling (NMDS) ordination of species composition of seed banks and plant community in wetlands with 0, 5, and 10 cm water levels on the Tibetan Plateau. Data for each wetland (T =  Typical wetland; D =  Drying wetland; S =  Saline-alkaline wetland) were analyzed separately. In addition, the seed banks and plant community of three wetlands were compared among each other (bottom-right). Ordination was based on relative abundance data. Different symbols represent different seed bank and plant community types. There were five sites for each seed bank, 10 plots for typical and saline-alkaline wetland plant communities and 6 plots for the drying wetland plant community. Location of ordination points within each diagram indicates degree of similarity between each one.

Overall, we identified 60 species belonging to 16 families in three wetlands and two species were unknown species. There were 25 species in typical wetland, 41 in drying wetland, and 14 in saline-alkaline wetland. Of these species, only one species (*Euphorbia helioscopia*) from typical wetland was annuals; all other species were perennials (Fig. 1). Species richness per quadrat differed significantly among three wetlands (Fig. 2), with the highest number in drying wetland and the lowest in saline-alkaline wetland. NMDS ordination showed the species composition of the plant communities have an obvious difference among the three wetlands (Fig. 3).

### Soil seed bank changes

**Figure 4:**
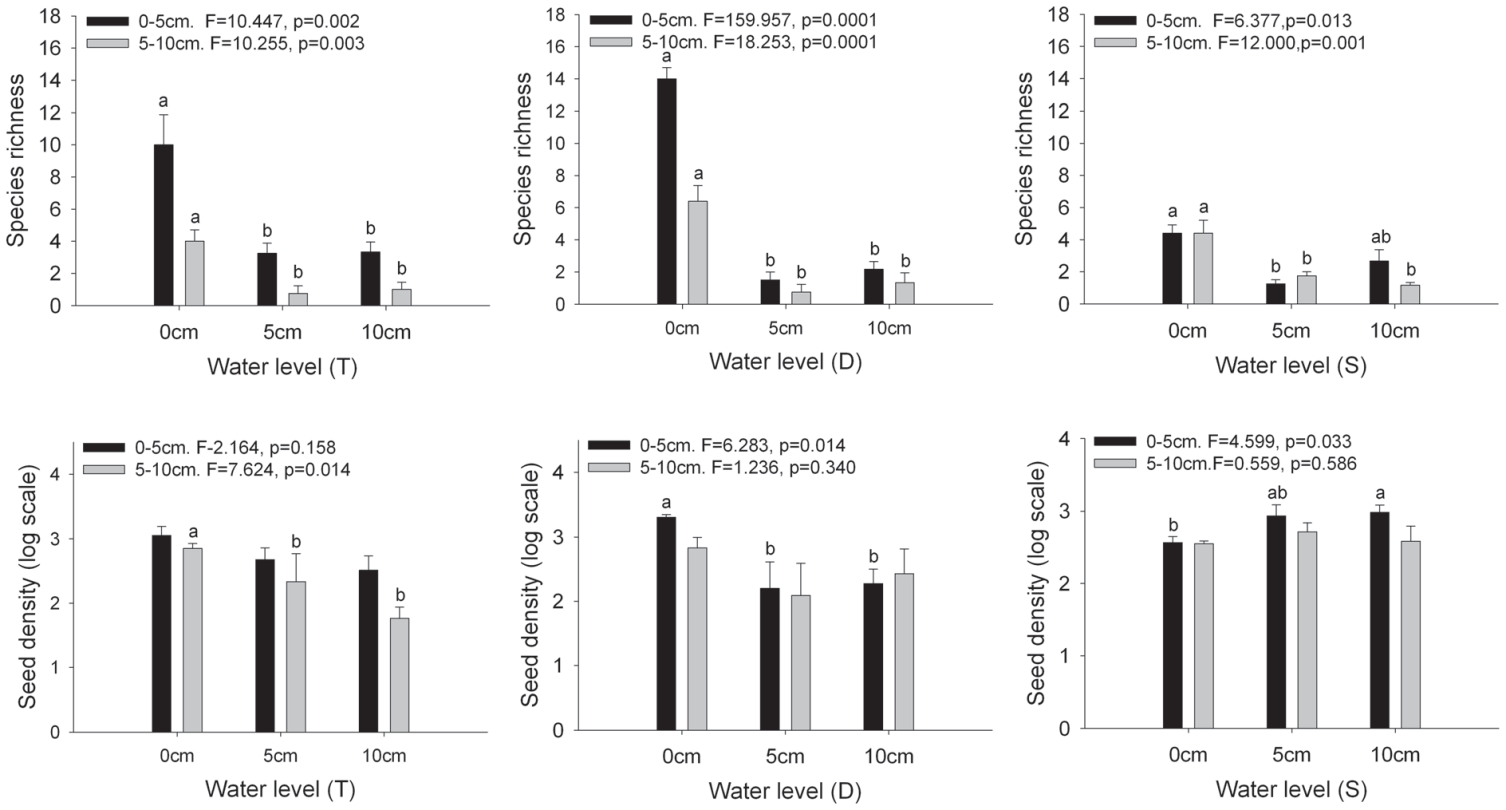
Species richness and seed density of 0–5 and 5–10 cm soil depths per plot (n = 5) changes in 0 cm, 5 cm and 10 cm water levels in three habitats. Seed density comparisons were carried out using log-transformed data. Different letters indicate significant differences (ANOVA, Tukey range test) of mean values of each soil depth. T =  Typical wetland; D =  Drying wetland; S =  Saline-alkaline wetland.

No seedlings emerged in the control trays. Overall, 1558 seedlings representing 57 species and 17 families emerged from all seed bank samples. Of these, 21.8 % were annuals, 3.6% were biennials, and 74.6% were perennial herbs. Two species could only be identified to the family level (Gramineae sp and Cyperaceae sp), and two could not be indentified at all. Species richness differed significantly among three wetlands, it was highest in drying wetland and lowest in saline-alkaline wetland (Fig. 4).

Proportion of perennial species germinated from seed bank was relative lower in 0 cm water level than 5 cm and 10 cm water level in all three wetlands (Fig. 1). Mean number of species richness per plot differed significantly among the three water levels in both the shallow and deep layers, showing a decrease trend with water level increase (Fig. 4). Seed density per plot differed significantly only in shallow layer in saline-alkaline wetland and drying wetland and only in the deep layer in wetland (Fig. 4), and there was no difference among different water levels in the other soil layers and wetlands. There no obvious trend showed in seed bank change in different wetlands: seed density increased with water level increase in the shallow layer in saline-alkaline wetland and decreased in shallow layer in drying wetland and in the deep layer in typical wetland.

### Similarity between seed bank and vegetation in different water level

In typical wetland, the 0 cm water level group clustered together, whereas the 5 cm and 10 cm water level groups had a scattered distribution (Fig. 3). The seed bank groups (0 cm, 5 cm, and 10 cm) were separated by dim 1 and 2 from the vegetation groups (Fig. 3). In drying wetlands, the 0 cm water level group clustered together. This group is closer to vegetation group from Dim 1 and 2. The 5 cm and 10 cm water level groups showed a scattered distribution and was far from vegetation group. In saline-alkaline wetlands, 5 cm and 10 cm water level groups overlapped with each other, they obvious differ with 0 cm group. Dim 1 and 2 axes separated the seed bank groups (0 cm, 5 cm, and 10 cm) from the vegetation groups (Fig. 3). Overall, the three vegetation groups were separated well by dim 1 but the seed bank groups overlapped with each other. The seed bank groups of saline-alkaline wetland and drying wetland were closer to their vegetation groups relative to the typical wetland seed bank group and its vegetation group (Fig. 3).

## Discussion

### Soil seed bank changes

Species richness was significantly higher in drying wetland than in the other two wetlands at the same water level and soil depth. We know the balance between seed input and output determines seed bank size [27]. In our study, species richness of vegetation was significant higher in drying wetland than typical wetland and saline-alkaline wetland (Fig. 2), and so this situation reflects in seed bank.

[7] found that submerged species dominate the seed bank community when the sediments were flooded for most of the time, while terrestrial species dominated the seed bank community when the sediments underwent rapid drying. However, we found that both the terrestrial species (alpine meadow species) and typical wetland species (semi-terrestrial and submerged species) dominated the seed banks of three wetlands. [2] also reported that there was no change in species composition of seed bank after alpine wetland drying and salinization. Species composition changed very little with alpine wetland plant community succession in the Tibetan Plateau [1].

Germination of wetland species from the seed bank may be determined by water level [9]–[10]. [28] found higher percentages of seed germination and seedling establishment in moist soil than in waterlogged soil, and no seedlings were established in flooded conditions. However, [29] found that different flooding regimes have no effect on the species composition of seed banks. We found that species richness was significantly higher in 0 cm water level than in 5 cm and 10 cm water levels, and there was no difference between 5 cm and 10 cm water level in each wetland and soil layer. Based on our results, we can conclude that the total number of species germinating decreased in static water level (5 cm and 10 cm water level) in our study. This is consistent with the results of several other studies [3], [17], [30]. These results are probably can be explained as follows. Germination and establishment of a species may occur in suitable environment conditions. In this study, we found that alpine meadow species make up a large component of the seed banks in all three wetlands, also including some other typical wetland species. Alpine meadow species germinated better under moisture conditions (0 cm water level), and wetland species germinated better under 5 cm and 10 cm water levels. Species germinated from 0 cm water level were alpine meadow species (Appendix S1). Seedlings from 5 cm and 10 cm water levels included only some typical wetland species, semi-terrestrial species (e.g. Cyperaceae sp, *Juncus bufonius*, *Arenaria serpyllifolia*, and *Blysmus sinocompressus*) and submerged species (*Utricularia vulgaris* and *Batrachium bungei*). Inundation suppressed germination of alpine meadow species from the seed bank in static water level (5 cm and 10 cm water level). Hence, more species germinated in 0 cm water level than static water levels.

Oxygen is necessary for seed germination and seedling growth. Light and oxygen levels are lower in flooded than in non-flooded conditions, thus the survival rate would be low in flooded conditions. Much research has found highest seed density in moist soil [3]. However, in our study, there is no obvious trend in seed density with different water levels, soil depths and wetlands. This can be explained as follows. [9] found that submersed, emergent and upland species germinated in all water levels, and not show an obvious trend. However, we found that the alpine meadow and typical wetland species germinated strictly in accordance with the expected water level treatments. All the alpine meadow species in the seed bank germinated in 0 cm water level (Appendix S1), e.g. *Artemisia desertorum*, *Taraxacum mongalicum*, *Plantago asihica*, *Humata tyermanni*, *Stipa przewalskyi*, and *Artemisia sieversiana* dominated the germinated plant community, and no typical wetland species germinated in this level. Meanwhile, we found Cyperaceae sp, *Arenaria serpyllifolia*, *Batrachium bungei*, *Utricularia vulgaris*, *Carex meyeriana*, and *Halerpestes cymbalaria*, which are typical wetland species, germinated only in flooded conditions (5 cm and 10 cm water levels). Species richness of wetland species was lower than alpine meadow species in 0 cm water level, but for these species, many seeds germinated in flooded condition. [31] found that anoxic conditions and constant high groundwater levels are favorable to the survival of seeds of *Juncus*. We also found that many *Juncus bufonius* seeds germinated in 5 cm and 10 cm water levels. In addition, water depth had little effect on germination of *Potentilla anserine* and *Carex moorcroftii*. These two species occurred under all of the three water levels, and has a wide regeneration niche.

### Relationship between species composition of seed bank and plant community in different water levels and importance for wetlands restoration

We found that the similarity of species composition between seed bank and plant community in 0 cm water level was higher in drying wetlands than the other two wetlands. The drying wetland is a transition stage between alpine wetland and alpine meadow during the gradual drying of the wetland. [1] found the similarity between seed bank and vegetation in alpine meadow is higher than that in alpine wetland. No waterlogged condition in drying wetlands is more conducive to seed germination and seedling survival. Wetlands species dominated in typical wetland. Many species from aboveground wetland plant community are being poorly represented in the seed bank, such as *Kobresia graminifolia*, *Potentilla anserine*, *Sanguisorba filiformis*, and *Caltha palustris*. The species that germinated from the seed bank in the 0 cm water level are alpine meadow species, so the similarity between seed bank and plant community is low in this wetland. Species composition of the saline-alkaline wetland plant community differs from that of typical and drying wetlands (Fig. 3). Dominant species in the saline-alkaline wetland are mostly clonal perennials that make a low contribution to the seed bank [2]. Some species we found in the vegetation but were absent in the seed bank, such as *Potentilla anserine*, *Halerpestes cymbalaria*, *Triglochin maritimum*, and *Pedicularis rhinanthoides*. In addition, high salinity in this wetland decreased species richness and abundance of plants that germinated from the seed bank. The aboveground plant community was dominated by some wetland species, which can tolerate high salinity environment in saline-alkaline wetland, e.g. *Kobresia capillifolia*, *Blysmus sinocompressus*, *Carex meyeriana*, and *Pedicularis rhinanthoides*. However, the species germinated in the 0 cm water level are alpine meadow species, such as *Taraxacum mongalicum*, *Plantago asihica*, *Artemisia desertorum*, and *Artemisia sieversiana*. Hence, the similarity between seed bank and plant community also is low in this wetland.

The similarity between plant community and seed bank is much lower in 5 cm and 10 cm water levels than 0 cm water level in all three wetlands. The plant community of all three wetlands that germinated from 5 cm and 10 cm water levels were dominated by submerged (*Utricularia vulgaris* and *Batrachium bungei*) and semi-terrestrial (*Cyperaceae sp*, *Halerpestes cymbalaria*, *Arenaria serpyllifolia* and *Juncus effusus*) species that were almost completely absent in wetland plant communities. Especially, many fewer species germinated in 10 cm water level. The following wetland species germinated only in 10 cm water level: submersed (*Utricularia vulgaris*) and semi-terrestrial species (Cyperaceae sp) in typical wetland, semi-terrestrial species (*Arenaria serpyllifolia*, Cyperaceae sp, *Halerpestes cymbalaria*, and *Juncus effuses*) in drying wetland, and submersed (*Batrachium bungei*) and semi-terrestrial species (*Arenaria serpyllifolia*, Cyperaceae sp, *Potentilla anserine*) in saline-alkaline wetland.

Species composition of the alpine wetland plant community has a huge change after drying and salinization of wetland. However, species composition of seed bank hardly changed whatever regardless of the environment change (drying and salinization of wetland). Alpine wetland species make up a large quantity of seeds in seed banks in all three wetlands, such as many typical wetland species, e.g. *Juncus bufonius*, *Blysmus sinocompressus*, *Kobresia littledalei*, *Scirpus setaceus*, and Cyperaceae sp. Especially, seed banks in three wetlands also are composed of characteristic alpine submerged species (*Utricularia vulgaris*, *Batrachium bungei*), and these species have a large number of seeds in seed bank. Presence of wetland species in the soil seed bank facilitate restoration [3]. Our study showed that target alpine wetland species for restoration remain in seed bank after restoration drying and salinization of alpine wetlands. Hence, lack of seeds and species is not a limited issue in the process restoration of the degraded wetlands. Further, we can conclude that the seed bank is an important potential resources for degraded wetlands restoration. Successful restoration of drying and salinization wetlands could depend on the seed bank.

Much research has concluded that hydrology is crucial for seed bank recruitment and vegetation establishment [10], [32]. We found that the water level is a major determinant of the final species composition of wetland plant communities. Further, different water levels in restored habitats is likely to determine species composition of plant communities. If allowing water level in moist situation during the spring may result in alpine meadow species, amphibious species and few wetland species germinated from the seed bank. Only submerged species would germinate if the water level more than 5 cm water level. The findings could provide further insight into how to restore the degraded alpine wetlands on the Tibetan Plateau.

## Supporting Information

Appendix S1 Seeds per m^−2^ of species in soil seed banks of the three wetland communities.
(DOC)
