# Supplementary material for: Effects of Water Level on Three Wetlands Soil Seed Banks on the Tibetan Plateau
Source: PLoS One. 2014 Jul 1;9(7):e101458. doi: 10.1371/journal.pone.0101458 (PMC4077783; doi:10.1371/journal.pone.0101458)
Supplement: Appendix S1 — Seeds per m−2 of species in soil seed banks of the three wetland communities. (DOC) [file pone.0101458.s001.doc]

**Appendix 1**. Seeds per m^-2^ of species in soil seed banks of the three wetland communities.

| **Species name** | **Typical wetland** | | | | | | **Drying up wetland** | | | | | | **Saline-alkaline wetland** | | | | | |
| --- | --- | --- | --- | --- | --- | --- | --- | --- | --- | --- | --- | --- | --- | --- | --- | --- | --- | --- |
|  | **0-5cm** | | | **5-10cm** | | | **0-5cm** | | | **5-10cm** | | | **0-5cm** | | | **5-10cm** | | |
|  | **0cm** | **5cm** | **10cm** | **0cm** | **5cm** | **10cm** | **0cm** | **5cm** | **10cm** | **0cm** | **5cm** | **10cm** | **0cm** | **5cm** | **10cm** | **0cm** | **5cm** | **10cm** |
| *Aconitum gymnandrum* |  |  |  |  |  |  |  |  |  |  |  |  | 39.3 |  |  |  |  |  |
| *Aellenia bryoniaefolium* |  |  |  |  |  |  | 78.5 |  |  |  |  |  |  |  |  |  |  |  |
| *Arenaria serpyllifolia* |  |  | 78.5 |  | 39.3 |  |  | 1884.8 | 981.7 |  |  | 78.5 |  | 78.5 | 706.8 |  | 549.7 | 157.1 |
| *Artemisia desertorum* | 117.8 |  |  | 549.7 |  |  | 1806.3 |  |  | 2591.6 |  |  | 2159.7 |  |  | 2199.0 |  |  |
| *Artemisia hedinii* |  |  |  | 39.3 |  |  | 157.1 |  |  |  |  |  |  |  |  |  |  |  |
| *Artemisia sieversiana* | 78.5 |  |  | 157.1 |  |  | 78.5 |  |  | 78.5 |  |  | 157.1 |  |  | 157.1 |  |  |
| *Batrachium bungei* |  |  |  |  |  |  | 1295.8 |  |  |  |  |  |  | 117.8 | 39.3 |  | 39.3 |  |
| *Batrachium capillaceus* |  |  |  |  |  |  |  |  |  | 117.8 |  |  | 431.9 |  |  |  |  |  |
| *Blysmus sinocompressus* |  |  | 981.7 |  |  |  |  |  |  |  |  |  |  |  |  |  |  |  |
| *Capsella bursa-pastoris* |  |  |  |  |  |  | 117.8 |  |  |  |  |  |  |  |  |  |  |  |
| *Carex meyeriana* |  |  | 78.5 |  |  |  |  |  |  |  |  |  |  | 117.8 |  |  |  |  |
| *Carex moorcroftii* |  |  |  | 39.3 |  |  | 314.1 |  |  | 157.1 |  |  | 471.2 |  | 39.3 | 78.5 |  |  |
| *Chenopodium iljinii* | 39.3 |  |  |  |  |  |  |  |  |  |  |  |  |  |  |  |  |  |
| *Chenopodium prostratum* |  |  |  |  |  |  | 39.3 |  |  |  |  |  |  |  |  |  |  |  |
| *Cirsium setosum* | 39.3 |  |  |  |  |  |  |  |  |  |  |  | 39.3 |  |  | 39.3 |  |  |
| *Corispermum stauntonii* | 39.3 |  |  | 39.3 |  |  |  |  |  | 78.5 |  |  |  |  |  |  |  |  |
| *Cynoglossum zeylanicum* |  |  |  |  |  |  | 39.3 |  |  |  |  |  |  |  |  |  |  |  |
| *Cyperaceae* sp |  | 1020.9 | 903.1 |  | 431.9 | 235.6 |  | 746.1 | 667.5 |  |  | 235.6 |  | 1060.2 | 1767.0 |  | 117.8 | 39.3 |
| *Elymus dahuricus* |  |  |  |  |  |  | 196.3 |  |  |  |  |  | 157.1 |  |  |  |  |  |
| *Festuca ovina* |  |  |  |  |  |  | 39.3 |  |  |  |  |  |  |  |  |  |  |  |
| *Fragaria orientalis* |  |  |  |  |  |  |  |  |  |  |  |  | 117.8 |  |  |  |  |  |
| *Gentiana dahurica* |  |  |  |  |  |  | 39.3 |  |  |  |  |  |  |  |  |  |  |  |
| *Geranium sibiricun* |  |  |  | 39.3 |  |  | 39.3 |  |  | 39.3 |  |  |  |  |  |  |  |  |
| *Geum aleppicum* |  |  |  |  |  |  |  |  |  |  |  |  |  | 39.3 |  |  |  |  |
| *Gramineae* sp |  |  |  |  |  |  |  |  |  |  |  |  | 78.5 |  |  |  |  |  |
| *Halerpestes cymbalaria* |  |  |  |  |  |  |  |  | 39.3 |  |  | 39.3 |  | 78.5 |  |  |  |  |
| *Humata tyermanni* | 392.7 |  |  | 39.3 |  |  | 157.1 |  |  | 196.3 |  |  | 39.3 |  |  | 157.1 |  |  |
| *Juncus bufonius* |  | 39.3 | 78.5 |  |  |  |  |  |  |  | 353.4 | 3377.0 | 39.3 | 314.1 | 117.8 |  |  |  |
| *Kobresia bellardii* |  |  |  |  |  |  |  |  |  |  |  |  |  |  |  |  |  |  |
| *Kobresia capillifolia* | 39.3 |  |  |  |  |  |  |  |  |  |  |  |  |  |  |  |  |  |
| *Kobresia graminifolia* |  |  |  |  |  |  |  |  |  |  |  |  |  |  |  |  |  |  |
| *Kobresia humilis* | 78.5 |  |  | 39.3 |  |  | 39.3 |  |  |  |  |  | 39.3 |  |  |  |  |  |
| *Kobresia tibetica* |  |  |  |  |  |  |  |  |  |  |  |  |  |  |  |  |  |  |
| *Leontopodium leontopodioides* |  |  |  |  |  |  |  |  |  |  |  |  | 39.3 |  |  |  |  |  |
| *Medicago ruthenica* |  |  |  |  |  |  |  |  |  | 39.3 |  |  |  |  |  |  |  |  |
| *Melilotus suaveolens* | 39.3 |  |  | 39.3 |  |  | 39.3 |  |  | 78.5 |  |  | 39.3 |  |  |  |  |  |
| *Myosoton aquaticum* |  |  |  |  |  |  | 39.3 |  |  |  |  |  | 157.1 |  |  |  |  |  |
| *Plantago asihica* | 39.3 |  |  | 117.8 |  |  | 2356.0 |  |  | 157.1 |  |  | 117.8 |  |  | 549.7 |  |  |
| *Poa annua* |  |  |  |  |  |  | 235.6 |  |  | 39.3 |  |  | 39.3 |  |  |  |  |  |
| *Poa crymophila* |  |  |  |  |  |  | 39.3 |  |  |  |  |  |  |  |  |  |  |  |
| *Poa declinata* |  |  |  |  |  |  | 431.9 |  |  |  |  |  |  |  |  |  |  |  |
| *Poa pachyantha* |  |  |  |  |  |  | 392.7 |  |  | 39.3 |  |  | 39.3 |  |  |  |  |  |
| *Poa poophagorum* |  |  |  |  |  |  | 157.1 |  |  | 39.3 |  |  |  |  |  |  |  |  |
| *Poa pratensis* |  |  |  |  |  |  | 274.9 |  |  |  |  |  |  |  |  |  |  |  |
| *Polygonum nepalense* |  |  |  |  |  |  |  |  |  | 39.3 |  |  |  |  |  |  |  |  |
| *Potentilla anserine* | 39.3 |  |  |  |  |  | 392.7 |  | 39.3 | 117.8 |  |  | 471.2 | 78.5 | 39.3 |  |  | 39.3 |
| *Potentilla bifurca* |  |  |  |  |  |  |  |  |  | 78.5 |  |  |  |  |  |  |  |  |
| *Potentilla fragarioides* |  |  |  |  |  |  | 235.6 |  |  |  |  |  | 39.3 |  |  |  |  |  |
| *Rumex acetosa* |  |  |  |  |  |  | 78.5 |  |  |  |  |  |  |  |  |  |  |  |
| *Scirpus distigmaticus* | 117.8 |  |  |  |  |  |  |  |  |  |  |  |  |  |  |  |  |  |
| *Scirpus setaceus* |  |  |  | 39.3 |  |  | 39.3 |  |  |  |  |  | 589.0 |  |  |  |  |  |
| *Senecio dubitabilis* |  |  |  |  |  |  | 39.3 |  |  | 39.3 |  |  | 117.8 |  |  |  |  |  |
| Sp1 |  |  |  |  |  |  |  |  | 196.3 |  | 78.5 | 117.8 |  | 274.9 | 117.8 |  |  | 39.3 |
| sp2 |  |  |  |  |  |  |  |  |  |  |  |  |  |  |  |  |  |  |
| *Stipa przewalskyi* | 39.3 |  |  |  |  |  | 117.8 |  |  |  |  |  | 942.4 |  |  |  |  |  |
| *Taraxacum mongalicum* | 863.9 |  |  | 667.5 |  |  | 1020.9 |  |  | 353.4 |  |  | 746.1 |  |  | 589.0 |  |  |
| *Utricularia vulgaris* |  | 4162.4 | 3259.2 |  | 2591.6 | 2316.8 |  | 196.3 |  |  |  |  |  |  |  |  |  |  |
